# Targeting IGF2BP3 in Cancer: From Molecular Structure and Biology to Early Drug Discovery

**DOI:** 10.3390/ijms27156992

**Published:** 2026-08-04

**Authors:** Elisa Uliassi, Maria Laura Bolognesi, Katia Scotlandi, Caterina Mancarella

**Affiliations:** 1 Dipartimento di Farmacia e Biotecnologie, Alma Mater Studiorum–Università di Bologna, 40126 Bologna, Italy; elisa.uliassi3@unibo.it (E.U.); marialaura.bolognesi@unibo.it (M.L.B.); 2 Laboratory of Oncology Research and Functional Genomics (ONCOGEN), IRCCS Istituto Ortopedico Rizzoli, 40136 Bologna, Italy

**Keywords:** RNA-binding proteins, IGF2BP3, RNA dynamics, epitranscriptomics, IGF2BP–RNA small molecule inhibitors, BET modulators, natural compounds, rigosertib, trabectedin

## Abstract

RNA-binding proteins (RBPs) remain underexplored as small-molecule targets, although their dysregulation contributes to numerous human diseases, including cancer. RBPs are key regulators of post-transcriptional gene expression, controlling multiple stages of RNA metabolism. Among them, insulin-like growth factor 2 mRNA-binding protein 3 (IGF2BP3) is an oncofetal RBP that is highly expressed during embryonic development, largely absent in adult tissues, and re-expressed in multiple malignancies. A growing body of evidence supports IGF2BP3 as a diagnostic and prognostic biomarker and a potent oncogenic driver across tumor types, underscoring its potential as a therapeutic target. However, the development of effective IGF2BP3-targeting compounds remains in its early stages. In this review, we first describe the structural organization of IGF2BP3, the molecular basis of RNA recognition, and the mechanisms underlying its dysregulation across human cancers. We then discuss emerging therapeutic approaches, including direct inhibition of IGF2BP3–RNA interactions and indirect strategies that rewire IGF2BP3 expression or activity through epigenetic, epitranscriptomic, and signaling pathways. By critically highlighting the opportunities and limitations of these approaches and their impact on cancer progression, we provide an integrated perspective combining structural biology, medicinal chemistry, and cancer biology to support the development of next-generation IGF2BP3-targeted therapies.

## 1. Introduction

RNA-binding proteins (RBPs) are critical post-transcriptional regulators of gene expression that control all stages of RNA metabolism—including transcription, splicing, transport, stability, and translation—and contribute to membraneless organelle formation and intracellular signaling networks. Large-scale studies have identified approximately 4200 RBPs in humans, reflecting a remarkable structural and functional diversity [1]. Through their ability to bind both messenger RNAs (mRNAs) and different types of non-coding RNAs, RBPs orchestrate critical physiological processes, tuning cell-fate decisions. Accordingly, dysregulation of RBPs is implicated in numerous diseases, including cancer [1]. Integrated molecular and genomic analyses have led to the identification of at least 16 families of RBPs annotated as oncogenic in human tumors, spanning from leukemia to carcinomas and sarcomas. While biological and molecular studies have provided roadmaps for their mechanisms of action and target genes, RBPs have not been fully explored as candidate targets in cancer therapy. As central post-transcriptional regulators controlling the fate of numerous RNA transcripts, these proteins represent attractive therapeutic targets for the simultaneous modulation of multiple oncogenic pathways. Pharmacological targeting of RBPs remains challenging due to intrinsically disordered regions, conserved domain homology, and complex interaction networks. Nevertheless, several RBP-targeting agents have shown promising results in preclinical and clinical studies. Inhibitors of the Musashi family [2] and HuR [3] reduced tumor cell survival and migration both in vitro and in vivo in solid and hematological malignancies. In addition, therapeutic strategies targeting FTO [4], SF3B1 [5], RBM39 [6], and nucleolin [7] have progressed toward phase I/II clinical evaluation in different cancer settings, showing preliminary evidence of feasibility and manageable safety profiles, despite clinical efficacy remaining to be fully established.

Among cancer-associated RBPs, the insulin-like growth factor 2 mRNA-binding protein 3 (IGF2BP3) has emerged as a major regulator of cancer-associated RNA networks and as an attractive therapeutic target.

IGF2BP3 is a member of the IGF2BP family of RBPs, which comprises three paralogs—IGF2BP1, IGF2BP2, and IGF2BP3—collectively known as IMPs or VICKZs [8,9,10,11]. They are named IGF2BPs because the three RBPs were initially identified as major post-transcriptional regulators of the growth factor IGF-2 during late mammalian development [12]. Subsequent studies established that IGF2BPs are evolutionarily conserved multidomain RBPs involved in the post-transcriptional regulation of transcripts controlling development, cell migration, metabolism, and tissue homeostasis [13]. More recently, IGF2BPs have been recognized as N6-methyladenosine (m^6^A) reader proteins, linking RNA modification-dependent regulation to physiological and pathological processes [13].

IGF2BP expression is tightly regulated during development. The three paralogs are produced in a burst at embryonic day 12.5 followed by a decline towards birth [12]. Studies in *Xenopus*, zebrafish, mice, and *Drosophila* have demonstrated conserved roles for IGF2BPs in cell-fate specification, embryonic patterning, tissue morphogenesis, and germline development through post-transcriptional regulation of developmental transcripts [14,15,16,17]. Following birth, expression of IGF2BP1 and IGF2BP3 is largely reduced. In human adult tissues, IGF2BP1 is detectable in bronchi, kidneys, ovaries, and testes, whereas IGF2BP3 expression is restricted to placenta, lymph nodes, tonsils, and testes [18,19]. On the contrary, IGF2BP2 remains expressed in most tissues, serving a role in nutrient and energy metabolism [20,21]. De novo expression of IGF2BP1 and IGF2BP3 occurs in diverse cancers, earning themselves the label of bona fide oncofetal proteins. However, while IGF2BP1 has also been found to play a tumor-suppressive role in multiple cancers including metastatic breast cancer, gastric cancer, and leukemia [22,23,24,25,26], evoking a more context-dependent expression and function across tumor types, IGF2BP3 has been consistently associated with aggressive disease and poor prognosis in cancer.

The biology and molecular functions of IGF2BP family members have been comprehensively summarized in previous reviews [27,28,29]. Importantly, despite their highly conserved RNA-binding domains, the three paralogs are not functionally redundant but regulate distinct RNA targets and biological processes.

In this review, we focus on IGF2BP3, integrating current knowledge of IGF2BP3 biology with emerging drug discovery strategies for cancer therapy. We discuss the structural basis of RNA recognition, IGF2BP3-specific molecular features within the IGF2BP family, molecular mechanisms underlying oncogenic functions, and recent advances in pharmacological modulation, highlighting both current challenges and opportunities for translating IGF2BP3 biology into clinically actionable interventions.

## 2. IGF2BP3 Architecture, and Molecular Basis for RNA Recognition

**Figure 1:**
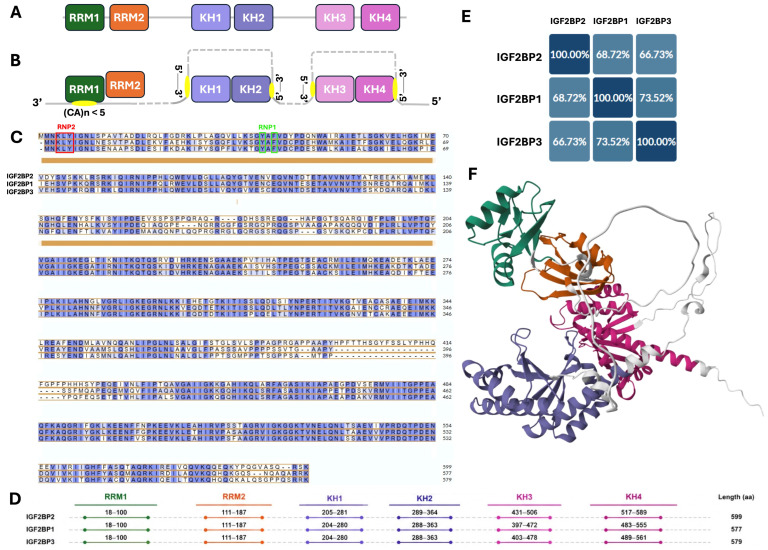
(**A**) General architecture of IGFBPs; (**B**) RRM1–RRM2 binds short CA-rich RNA, while KH domains mediate antiparallel RNA binding; (**C**) Clustal-colored sequence alignment of *Hs*IGFBP1, 2 and 3 highlighting conserved and variable regions; (**D**) Comparison of domain boundaries and lengths among IGF2BP1–3; (**E**) Percent identity matrix showing moderate-to-high sequence similarity among IGFBP1–3; (**F**) AlphaFold structural model of IGFBP3 with domains color-coded: RRM1 (green), RRM2 (orange), KH1–KH2 (violet), and KH3–KH4 (magenta).

IGF2BPs family represents a prototypical example of multidomain RBPs and is characterized by a common architecture of six RNA-binding units: two N-terminal RNA-recognition motifs (RRMs) and four consecutive heterogeneous nuclear ribonucleoprotein (hnRNP) K-homology (KH) domains, each contributing to a different extent to RNA binding (Figure 1A–D). The sequence alignment performed by using the Clustal Omega algorithm 1.2.4 of UniProt [30] shows that IGF2BP1, IGF2BP2, and IGF2BP3 share moderate-to-high sequence identity (Figure 1C), consistent with the identity matrix (≈63–74%, Figure 1E). Despite differences in the overall protein length, the domain organization and domain boundaries are remarkably conserved among the three paralogs, with nearly identical positions of the RRM and KH domains, underscoring the strong evolutionary conservation of their RNA-binding architecture (Figure 1D). The highest identity between IGF2BP1 and IGF2BP3 (~73.5%) is reflected in their closer similarity in alignment. Conserved regions, particularly within the central and C-terminal portions, point to a shared structural core and common functional domains. The RRM1 and RRM2 domains are connected by a short linker sequence, suggesting a compact arrangement that may contribute to RNA-binding specificity. Structural analyses of IGF2BP3 show that CA-rich RNA is contacted by the exposed RRM1 β-sheet through Tyr5 in the RNP2 motif and Tyr39 and Phe41 in the RNP1 motif; the aligned homologous residues are Tyr5, Tyr39, and Phe41 in IGF2BP1 and IGF2BP3 and Tyr6, Tyr40, and Phe42 in IGF2BP2, whereas the RRM2 β-sheet is occluded and does not directly contact RNA in the solved RRM12–RNA complexes (Figure 1C) [31,32]. Similarly, KH1–2 and KH3–4 domains function as preorganized RNA-binding modules that bind bipartite RNA motifs [33]. In contrast, variable regions characterized by insertions and gaps (e.g., around residues 160–220 and ~400) likely represent flexible or regulatory segments that underlie key functional differences among IGF2BP family members [13].

To date, only a limited number of structures of IGF2BP–RNA complexes have been resolved, most of which involve individual domains, making it difficult to fully understand their specific contributions and dynamic interplay [34]. Notably, only in August 2025 was a full-length structural model of IGF2BP3 generated using AlphaFold [35,36], encompassing all domains (Figure 1F; see caption for domain color-coding). This advance, together with integrated approaches such as SELEX-seq, motif-spacing analyses, functional validation assays, and structural biology [33], offers valuable insights into the motif combinations and spacing preferences across IGF2BP1, 2 and 3. Despite these advances [37], the mechanisms by which multiple RNA-binding domains (RBDs) cooperate to achieve high selectivity and affinity, as well as the extent of flexibility in RNA–protein interaction patterns, remain only partially understood, largely due to the dynamic arrangements of multiple RBDs during RNA binding.

The high-resolution structure of IGF2BP3 RRM1–RRM2 tandem (RRM12, PDB: 6FQ1) reveals the canonical RRM fold, consisting of a four-stranded antiparallel β-sheet backed by two α-helices [31]. High-resolution structures of RNA-bound complexes (PDB IDs: 6FQR, 6GX6, 7YEW, 7YEX, and 7VSJ) [31,32] further demonstrate that RNA binding is functionally asymmetric, with only RRM1 directly engaging the RNA. Specifically, RRM1 binds CA-rich motifs through hydrogen bonding and base-stacking interactions, while RRM2 plays a structural role, likely stabilizing the compact RRM1–2 arrangement [31]. The noncanonical orientation of the RRM1–2 interdomain precludes the formation of a continuous RNA-binding surface, thereby restricting recognition to a minimal dinucleotide or short CA-rich motif (Figure 1B).

In contrast, the KH domains act as extended RNA-binding platforms. Structural analyses of the KH1/2 domains (PDB IDs 6GQE, 7YEY, 7VKL) [32,33] show that they form an antiparallel RNA-binding unit, in which the RNA strand is oriented antiparallel to the protein backbone. Within this tandem, KH1 accommodates a longer RNA segment (≈5 nucleotides), while KH2 binds a shorter element (≈2 nucleotides), forming a continuous binding interface (Figure 1B). This arrangement generates a cleft defined by the conserved GxxG loop, enabling recognition of extended CA-rich and structured RNA elements [32].

Although high-resolution structures of KH3–KH4 of IGF2BP3 are less extensively characterized, comparative analyses indicate that they share similar folds and likely contribute additional RNA contacts, hence reinforcing multivalent binding. KH3–KH4 domains can accommodate longer RNA sequences, such as the β-actin zipcode [32]. The repetition of KH modules supports a model of modular amplification, in which each domain incrementally enhances binding affinity and specificity [32]. Accordingly, RNA recognition by IGF2BP3 is governed by a hierarchical and multivalent mechanism.

Domain-resolved SELEX-seq and structural studies provide a more specific sequence-recognition code. The RRM1–RRM2 tandem preferentially binds extended CA-rich or CA-repeat elements, with direct contacts made mainly by RRM1. Both KH tandems recognize bipartite combinations of a GGC-core element and a CA-rich element. In IGF2BP3, KH1 preferentially contacts GGC, whereas KH2 favors CA-rich RNA. Motif-spacing analysis identified a preferred CA–N_22_–_25_–CGGC arrangement for KH1–KH2 and GGCA–N_17_–_25_–CA, together with related CGGC/CA orientations, for KH3–KH4. Thus, target selection depends on motif identity, order, and spacing rather than on a single short consensus [33]. Paralog-specific differences also occur: IGF2BP2 KH3 recognizes a UCA/CA element, whereas KH4 requires a GG-containing element, indicating that conserved KH folds can encode distinct sequence preferences through their variable loops. This results in a multivalent binding mode in which overall affinity arises from the cumulative effect of multiple weak interactions distributed along the RNA. Importantly, specificity is not dictated by a single consensus sequence but rather by the pattern, spacing, and density of CA-rich motifs compatible with the domain architecture. Integrated RIP, PAR-CLIP, and m^6^A-seq methods demonstrated that all three IGF2BPs bind their target RNA to the “UGGAC” consensus sequence containing the “GGAC” m^6^A core motif. Moreover, 92% of IGF2BP binding sites occur within protein-coding transcripts, and more than 80% of high-confidence targets contain at least one m^6^A peak, as identified by m^6^A-seq [38]. Recent evidence indicates that IGF2BPs directly interact with the RBP YBX1 promoting the stabilization and expression of m^6^A-modified transcripts [39]. Each paralogue binds approximately 4000 target RNAs, with a 55 to 70% high confidence shared targets [38]. Accordingly, IGF2BPs can interact with target RNAs as homo- or heterodimers, which may account, at least in part, for the shared RNA targets identified among family members [40].

Together, functional data and high-resolution structural analysis have provided valuable insights into the molecular determinants governing RNA recognition and highlight opportunities for targeting IGF2BP3 in anticancer drug discovery.

## 3. Distinctive Features of IGF2BP3 Among IGF2BP Paralogs

Although IGF2BP1, IGF2BP2, and IGF2BP3 share a highly conserved domain organization and substantial sequence identity, increasing evidence indicates that they have evolved distinct regulatory programs rather than redundant functions. Transcriptome-wide analyses estimate that approximately 30–50% of IGF2BP-regulated transcripts are uniquely controlled by individual paralogs, demonstrating that closely related RBPs can establish remarkably different post-transcriptional networks. Current evidence suggests that paralog specificity emerges through multiple hierarchical levels, including differences in RNA recognition, RNA-binding domain utilization, assembly into ribonucleoprotein (RNP) complexes, subcellular localization, and downstream regulation of mRNA fate.

Among the three paralogs, IGF2BP1 and IGF2BP3 are considered the most closely related based on amino acid sequence similarity, developmental expression patterns, and extensive overlap in CLIP-derived interactomes [41]. In contrast, IGF2BP2 exhibits more specialized physiological functions, particularly in metabolic regulation. Despite the close evolutionary relationship between IGF2BP1 and IGF2BP3, transcriptome-wide binding studies have revealed substantial differences in RNA target selection. Whereas IGF2BP1 and IGF2BP2 preferentially associate with the 3′ untranslated regions (UTRs) of mature transcripts, IGF2BP3 displays a pronounced enrichment for coding sequences, suggesting distinct mechanisms of transcript recognition and regulation [42]. In addition, although early CLIP studies and in vitro selection approaches (SELEX, RNAcompete and Bind-n-Seq) identified a general preference of the IGF2BP family for CA-rich RNA elements, subsequent structural and biochemical analyses demonstrated important paralog-specific differences. In contrast to the IGF2BP1-associated CGGAC motif, IGF2BP3 preferentially recognizes two related GGC-core motifs (GGCA and CGGC), whose binding depends not only on sequence identity but also on their relative spacing and cooperative interaction with adjacent CA-rich elements. In addition, recent in vitro analyses using recombinant RBPs and native RNA substrates indicate that paralog specificity reflects not only differences in RNA sequence recognition but also distinct balances between affinity, avidity and target selection. Accordingly, genetic variants of IGF2BP1 and IGF2BP3 predominantly modulate RNA-binding affinity without substantially altering target specificity, whereas the IGF2BP2 variants primarily change target selection while preserving overall binding strength [43].

Increasing evidence indicates that specificity also emerges from the dynamic organization of RNP complexes. In the cytoplasm, IGF2BP1 and IGF2BP3 assemble large, motile ribonucleoprotein granules known as locasomes, which function as repositories for translationally silent mRNAs [12]. These granules are depleted of ribosomes, translation initiation factors and most components of the RNA-induced silencing complex (RISC), thereby protecting associated transcripts from premature translation and degradation. Nevertheless, IGF2BP3 differs from IGF2BP1 in its ability to recruit RISC components to locasomes, suggesting that IGF2BP3 integrates both mRNA protection and selective miRNA-dependent regulation within the same ribonucleoprotein environment [44]. Consistent with this model, compartmentalization of transcripts into IGF2BP3-containing granules has been proposed as a mechanism limiting miRNA-mediated mRNA decay during tumor progression.

Recent work has also revealed an additional layer of functional divergence in translational control. Although all three IGF2BP proteins act as readers of m^6^A-modified transcripts, the downstream consequences of m^6^A recognition differ substantially among the paralogs. IGF2BP3 is enriched in processing bodies (P-bodies), where it associates with multiple translation repressors, including 4E-T. Rather than promoting translation, IGF2BP3 redirects its target transcripts from actively translating ribosomes into P-bodies, thereby repressing protein synthesis while maintaining transcript stability [45]. This mechanism contrasts with the predominantly mRNA-stabilizing activities generally attributed to IGF2BP1 and IGF2BP2 and further emphasizes the unique role of IGF2BP3 in dynamic translational regulation.

Spatial and temporal co-expression of RNA and protein, and interactions with additional RBPs collectively determine transcript accessibility and regulatory outcome. Unlike IGF2BP1 and IGF2BP2, IGF2BP3 displays a significant nuclear pool, where it associates with transcripts encoding Cyclin D1, Cyclin D3 and Cyclin G1. This nuclear localization depends on interaction with the RBP HNRNPM and is essential for efficient post-transcriptional regulation of cyclin expression. Disruption of the IGF2BP3–HNRNPM complex or its cytoplasmic retention markedly reduces proliferation of human cancer cells, highlighting a unique nuclear function of IGF2BP3 in cell-cycle control [46].

These features distinguish IGF2BP3 from the other IGF2BP paralogs and provide a mechanistic basis for its diverse biological functions. Accordingly, IGF2BP3 regulates a broad repertoire of target mRNAs involved in multiple biological processes (Table 1).

## 4. IGF2BP3 in Cancer: A Central Hub Linking Epitranscriptomic and Post-Transcriptional Regulation

IGF2BP3 is recurrently reactivated across diverse human malignancies, where it drives tumorigenesis and cancer progression. Accordingly, alterations in IGF2BP3 expression or function have implications for multiple cancer hallmarks, including increased cell proliferation [72], migration and invasion [47], therapy-resistant cellular states [73], vasculogenesis [71], apoptosis blockade [72], and epithelial–mesenchymal transition [74]. Additionally, emerging IGF2BP3-driven processes include metabolic reprogramming, such as activation of lipogenesis [75] and enhanced glycolysis [76], ferroptosis resistance [61], immune evasion [77], and polarization of tumor-associated macrophages toward an M2 phenotype [78].

Beyond its role in cancer progression, IGF2BP3 has also been proposed as a diagnostic and/or prognostic biomarker. Pan-cancer analyses have shown that approximately 16% of human tumors display higher IGF2BP3 expression compared with matched adjacent normal tissues. In addition, survival analyses across human cancers indicate that elevated IGF2BP3 expression is associated with significantly shorter disease-specific survival in approximately 40% of tumor types compared with low expression levels IGF2BP3 expression [79]. Previously published reviews have provided a detailed discussion of the specific roles of IGF2BP3 in different human cancers, including leukemia, brain tumors, carcinomas, and sarcomas, as well as the target RNAs involved in each malignant process [80,81]. Here, we synthesize the current understanding of IGF2BP3 expression deregulation and its molecular mechanisms of action in cancer, providing a framework to support therapeutic strategies aimed at inhibiting its function and thereby counteracting its pleiotropic oncogenic effects.

### 4.1. Mechanisms Underlying IGF2BP3 Dysregulation in Cancer

Mechanisms underlying the de novo expression of IGF2BP3 in cancer remain only partially understood and appear to be highly dependent on tumor-context, making the identification of a unifying regulatory mechanism particularly challenging. Proposed mechanisms include epigenetic rewiring, aberrant expression of transcriptional regulators, post-transcriptional feedback loops, and post-translational modulation of protein stability, whereas mutations affecting *IGF2BP3* are relatively uncommon. Gene amplification represents the most frequent genomic alteration involving *IGF2BP3* across cancer types, reaching the highest frequency in uterine carcinosarcoma (6%), and occurring at approximately 4% in uterine corpus endometrial carcinoma, skin cutaneous melanoma, stomach adenocarcinoma, and colon adenocarcinoma [79]. Somatic alterations, including missense mutations and single nucleotide polymorphisms, are comparatively rare, with the highest frequencies reported in liver cancer (0.54%) and colorectal cancer (1.52%) [82]. A notable exception is thyroid cancer, where *THADA–IGF2BP3* fusions, involving the *THADA* gene locus on chromosome 2 and a region on chromosome 7 immediately proximal to *IGF2BP3*, drive IGF2BP3 overexpression and are typically found in tumor entities with a clinically low-risk profile [83].

IGF2BP3 up-regulation in cancer has frequently been associated with epigenetic promoter hypomethylation [84,85,86,87]. Comprehensive DNA methylation sequencing studies across 15 major human cancer types identified a significant inverse correlation between methylation levels at the *IGF2BP3* promoter and *IGF2BP3* mRNA expression. Distinct CpG islands within the *IGF2BP3* promoter were found to be demethylated in triple-negative breast cancer and intrahepatic cholangiocarcinoma tissues compared with matched non-neoplastic tissues, in which these regions remained methylated [85,87]. In triple-negative breast cancer, systematic silencing of DNA demethylases identified TET3 as a major contributor to *IGF2BP3* promoter demethylation [85]. Consistently, pharmacological inhibition of DNA methyltransferases using decitabine induced IGF2BP3 expression at both mRNA and protein levels, further supporting a direct role for DNA methylation in regulating *IGF2BP3* transcription [86].

Aberrant activation of transcription factors also contributes to increased IGF2BP3 expression. IGF2BP3 has been identified as part of MYC-dependent transcriptional programs [88]. In neuroblastoma cells, ChIP-qPCR analyses demonstrated that MYCN directly binds the *IGF2BP3* promoter and enhances its transcriptional activity [88]. More recent evidence indicates that MYCN is embedded in a core transcriptional regulatory circuitry composed of interconnected autoregulatory factors associated with super-enhancers, which collectively sustain IGF2BP3 expression [89]. Consistently, ChIP-seq analyses revealed direct binding of MYCN, PHOX2B, HAND2, and GATA3 to the *IGF2BP3* promoter coinciding with prominent H3K27Ac enrichment and an active chromatin state [89]. Although evidence remains limited, ChIP experiments have also identified increased NF-κB p65 occupancy at the *IGF2BP3* promoter in glioblastoma cell lines [90]. In these models, IGF2BP3 in turn promotes p65 translation, establishing a positive feedback loop that contributes to glioblastoma cell migration and maintenance of stemness [90].

At the post-transcriptional level, accumulating evidence indicates that IGF2BP3 is extensively regulated by non-coding RNA networks. Multiple tumor-suppressive microRNAs (miRNAs) have been reported to directly bind the (3′ UTRs of *IGF2BP3* mRNA, thereby reducing its expression and impairing tumor cell proliferation, migration, invasion, and stemness-related phenotypes in different cancer models. These include members of the let-7 family [91], miRNA-34a [92], miRNA-129-1 [93], miRNA-375-3p [94], miRNA-654 [95], and miRNA-200a [96]. Conversely, oncogenic long non-coding RNAs (lncRNAs) and circular RNAs (circRNAs) sustain IGF2BP3 expression and activity through different mechanisms. In endometrial carcinoma, the lncRNA LINC00958 directly interacts with IGF2BP3 in the cytoplasm, enhancing its ability to stabilize target mRNAs [97]. Among circRNAs, circITGB6 interacts directly with IGF2BP3, increasing the stability of downstream target transcripts such as *PDPN*, a key effector of epithelial–mesenchymal transition (EMT) and metastasis [98].

Protein stability represents another critical determinant of IGF2BP3 activity and is tightly regulated by ubiquitination-dependent pathways. To date, the deubiquitinases USP10 and USP11 have both been shown to stabilize IGF2BP3 protein levels, preventing its proteasomal degradation and promoting tumorigenic properties in colorectal cancer and non-small cell lung cancer [74,99]. USP10 increases IGF2BP3 stability by removing K48-linked polyubiquitination chains, thereby inhibiting proteasome-mediated degradation [74]. Conversely, E3 ubiquitin ligases Parkin and MKRN2 promote IGF2BP3 turnover through site-specific ubiquitination, thereby modulating downstream oncogenic signaling pathways, including PI3K and MAPK signaling [55,100].

Interestingly, the major mechanisms governing IGF2BP3 expression are globally conserved across the IGF2BP family, as highlighted in the recent comprehensive review by Duan et al. [27]. Epigenetic regulation, transcriptional control, post-transcriptional regulation, and post-translational mechanisms all contribute to the regulation of IGF2BP1, IGF2BP2, and IGF2BP3. However, a closer analysis of the individual regulatory factors operating at each of these levels reveals only limited overlap among the three paralogs.

**Figure 2:**
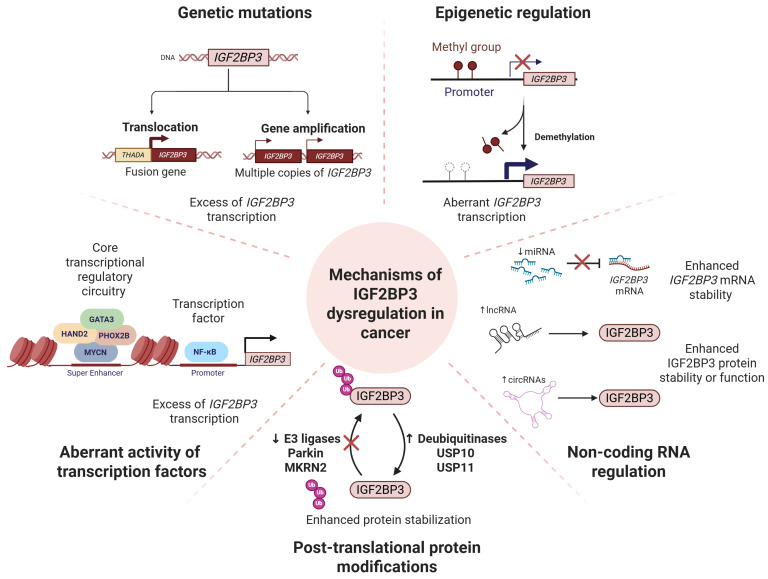
Schematic representation of the molecular mechanisms underlying IGF2BP3 dysregulation in cancer. *IGF2BP3* transcription is regulated by i. genetic alterations, including chromosomal translocation and gene amplification; ii. DNA demethylation of the *IGF2BP3* promoter; and iii. activation by transcription factors such as MYCN, members of its core transcriptional regulatory circuitry, and NF-κB. IGF2BP3 expression and function are also regulated by different classes of non-coding RNAs. MicroRNAs (miRNAs) negatively regulate *IGF2BP3* at the post-transcriptional level, and their downregulation leads to increased *IGF2BP3* expression, whereas lncRNAs and circRNAs enhance IGF2BP3 stability and activity. At the post-translational level, IGF2BP3 is regulated by the ubiquitin–proteasome system, in which E3 ligases and deubiquitinases dynamically control its proteasomal degradation. Created in BioRender. Mancarella, C. (2026) https://BioRender.com/229vbiv.

A schematic overview of the major molecular mechanisms underlying IGF2BP3 dysregulation in cancer is presented in Figure 2.

### 4.2. Mechanisms of IGF2BP3-Mediated Oncogenic Activity

One of the best-characterized functions of IGF2BP3 is its role as a reader of m^6^A, the most abundant internal mRNA modification in eukaryotes [101]. m^6^A is deposited by METTL3/METTL14-containing methyltransferase complexes and is enriched near stop codons and within 3′ UTRs [102]. As first demonstrated by Huang et al. [38], IGF2BP family members, including IGF2BP3, selectively bind m^6^A-modified transcripts and enhance their stability, thereby sustaining the expression of oncogenic factors such as MYC. This axis represents a central mechanism of IGF2BP3-driven tumorigenesis and is widely observed across cancer types [103]. Beyond direct mRNA stabilization, IGF2BP3 may also indirectly influence epitranscriptomic regulation by enhancing the translation of MAT2B, a component of the methionine adenosyltransferase complex involved in S-adenosylmethionine production [67].

In addition to m^6^A, emerging evidence indicates that IGF2BP3 can recognize additional RNA modifications. It binds N7-methylguanosine (m^7^G)-modified transcripts and promotes their degradation in cancer cells [70]. In glioblastoma, IGF2BP3 facilitates recruitment of m^7^G-marked mRNAs to the exosome complex through interaction with EXOSC2, accelerating RNA decay. Among these targets, *TP53* mRNA is regulated in an m^7^G-dependent manner, affecting transcript stability and chemosensitivity [70]. Collectively, these findings expand the functional repertoire of IGF2BP3 and support a context-dependent role in RNA fate determination.

A second major function of IGF2BP3 involves its tight interconnection with the miRNA machinery. IGF2BP3 protects target transcripts, such as *HMGA2* and *LIN28B*, from miRNA-dependent silencing by sequestering them within RNP complexes that are inaccessible to the RISC complex [44,81]. In addition, IGF2BP3 competes with AGO2–miRNA complexes for 3′ UTR binding, thereby modulating post-transcriptional gene silencing [104]. However, the mechanisms through which RISC is excluded from RNP granules, as well as the precise nature of the interaction between RISC and IGF2BP3, remain poorly understood. Beyond antagonizing miRNA activity, IGF2BP3 can also influence miRNA biogenesis. It directly interacts with Drosha in the nucleus, and the resulting IGF2BP3–Drosha complex processes specific pri-miRNAs, contributing to the generation of 3′-isomiRs from a subset of miRNAs [44]. In other contexts, IGF2BP3 has been implicated in suppressing miRNA maturation [105]. Through modulation of Drosha-dependent processing pathways, IGF2BP3 reshapes miRNA repertoires in cancer cells. Beyond microRNAs, IGF2BP3 also regulates transcript stability through interactions with lncRNAs and circRNAs. LncRNAs such as LINC01138 [106] enhance the stability of oncogenic transcripts through IGF2BP3, whereas linc-SPRY3 acts as a molecular decoy that limits IGF2BP3 availability [107]. Likewise, circRNAs modulate IGF2BP3 activity by serving as molecular scaffolds or through m^6^A-dependent interactions. Recent evidence include circRARS, which enhances IGF2BP3-mediated m^6^A recognition to drive renal cell carcinoma progression [108]. However, the molecular mechanisms governing these interactions and their context-specific functional consequences remain incompletely understood and warrant further investigation.

A third major function of IGF2BP3 relies on its association with protein partners. IGF2BP3 is a key component of cytoplasmic RNP granules, including processing bodies and stress granules, where it regulates the spatial and temporal control of mRNA metabolism. IGF2BP3-containing RNPs have been proposed to function as “cytoplasmic safe houses” that protect oncogenic transcripts from degradation [62]. In addition, IGF2BP3 promotes the sequestration of m^6^A-modified mRNAs into these compartments, thereby influencing their translational fate. Through interactions with proteins such as G3BP1 and LIN28A, IGF2BP3 contributes to stress granule assembly and cellular adaptation to stress conditions [109,110]. Moreover, IGF2BP3-containing RNP complexes undergo active microtubule-dependent transport toward specific subcellular regions, including the leading edge of migrating cells. This spatial regulation enables localized translation of transcripts involved in cytoskeletal remodeling and cell motility, thereby promoting invasive and metastatic phenotypes [50]. In pancreatic ductal adenocarcinoma, this process is mediated by the motor protein KIF20A, which regulates the trafficking of IGF2BP3-positive granules and supports membrane protrusion dynamics and cell invasion. Among IGF2BP3 protein partners, USP10 has been shown to deubiquitinate IGF2BP3. Interestingly, physical interaction between IGF2BP3 and USP10 can attenuate USP10-mediated deubiquitination of tumor suppressor targets such as p53 [111].

**Figure 3:**
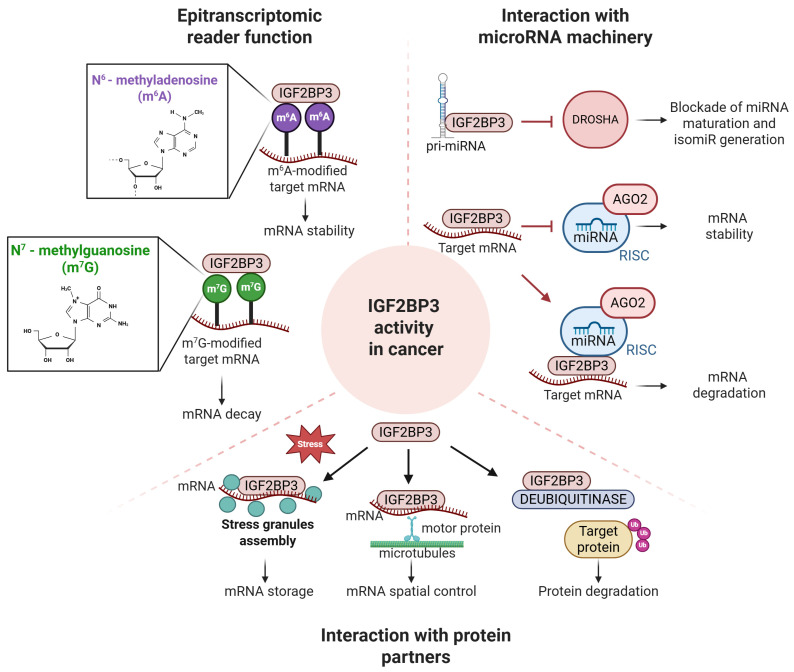
Schematic representation of IGF2BP3 activity in cancer. IGF2BP3 promotes tumor progression through three main functional axes: (i) epitranscriptomic regulation, acting as an m^6^A reader that stabilizes oncogenic transcripts, while also binding m^7^G-modified mRNAs to promote their degradation; (ii) modulation of microRNA pathways, by influencing microRNA biogenesis through interaction with Drosha, protecting target mRNAs from RISC-mediated silencing, and competing with AGO2 complexes for mRNA binding; and (iii) interaction with protein partners, including stress granule-associated proteins, motor proteins, and deubiquitinases, thereby regulating mRNA storage, microtubule-dependent transport to specific subcellular regions, and target protein stability, respectively. Created in BioRender. Mancarella, C. (2026) https://BioRender.com/e696phv.

A schematic representation of major functional axes of IGF2BP3 in cancer is shown in Figure 3.

## 5. Combining Molecular Insights and Chemical Strategies to Target IGF2BP3 in Cancer

Targeting IGF2BP3 has emerged as an important strategy in cancer drug discovery, given its central role in stabilizing oncogenic mRNAs through m^6^A-dependent recognition. In this section, we discuss therapeutic approaches that either directly disrupt IGF2BP3–RNA interactions or indirectly modulate its expression and functional activity in cancer. These strategies, described in Section 5.1 and Section 5.2, respectively, integrate key molecular insights from structural and functional studies, highlighting both opportunities and current limitations in drug development.

### 5.1. Disrupting IGF2BP3-RNA Targets Interactions Using Small Molecules

**Figure 4:**
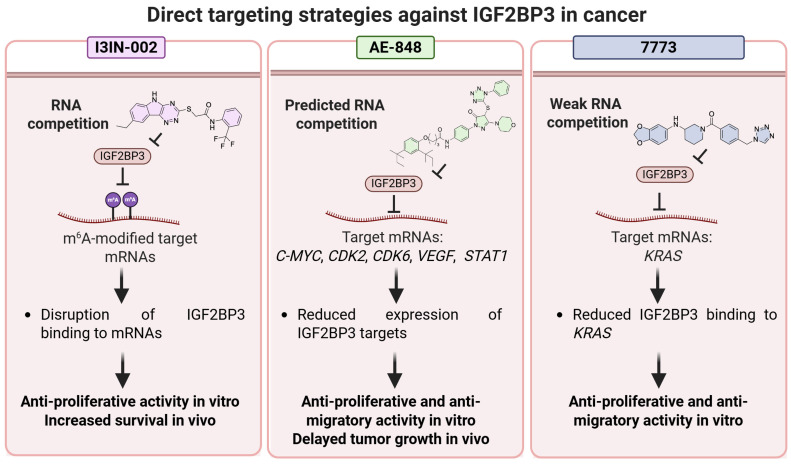
Schematic representation of direct small-molecule modulators targeting IGF2BP3 in cancer. Each panel shows compound structure, proposed effect on IGF2BP3 function, and biological responses relevant to cancer progression. I3IN-002 inhibits IGF2BP3–RNA interactions, reducing m^6^A-modified target expression and impairing IGF2BP3-dependent oncogenic programs. AE-848 modulates IGF2BP3-associated pathways, reducing expression of oncogenic targets and suppressing tumor cell proliferation, migration, and invasion, with in vivo anti-tumor effects. 7773 targets IGF2BP KH domains, partially inhibits RNA binding, and reduces tumor cell proliferation and migration in vitro. Created in BioRender. Mancarella, C. (2026) https://BioRender.com/r8xs9ki.

Direct targeting of IGF2BP3 remains particularly challenging due to the limited availability of high-resolution structural data and the inherently dynamic nature of IGF2BP–RNA interactions. Nevertheless, a small number of ligands have been experimentally validated as direct inhibitors of IGF2BP–RNA binding, while several others remain computationally predicted with limited structural and biochemical confirmation [112]. Consistent with these challenges, most currently available direct inhibitors exhibit only low-to-moderate binding affinity and therefore require micromolar concentrations to achieve measurable target engagement and disruption of IGF2BP3–RNA interactions in biochemical and cellular assays. Moreover, many reported compounds exhibit poor selectivity across IGF2BP family members, reflecting the high degree of structural conservation among their RNA-binding domains. Achieving IGF2BP3-selective inhibition will require exploiting subtle paralog-specific differences in sequence composition, conformational dynamics, and ligandable surface features within RNA-binding domains, supported by high-resolution structural studies and structure-guided medicinal chemistry approaches. These subtle sequence and conformational differences are difficult to exploit, posing a significant barrier to the rational design of truly selective IGF2BP3 inhibitors. Direct chemical targeting of IGF2BP3 remains highly challenging due to structural plasticity and limited high-resolution ligand–protein data. Future progress will depend on integrating structural biology, biophysics, and medicinal chemistry to develop potent and selective IGF2BP3 inhibitors. A schematic representation of the compounds, which will be discussed below, is shown in Figure 4.

#### 5.1.1. I3IN-002: Early Proof-of-Concept for Pharmacological IGF2BP3 Inhibition

I3IN-002 was identified by Rao et al. through a structure-based virtual screening strategy targeting the RRM12 domain of IGF2BP3 (PDB ID: 6GX6), followed by TR-FRET and cell-based assays [113,114]. The compound was selected from 417 hits based on activity of the indolyltriazine scaffold and further validated in SEM leukemia cells expressing IGF2BP3 (SEM-WT), with reduced activity observed in IGF2BP3 knockout cells (SEM-I3KO). Both the thermal shift assay (TSA) and cellular thermal shift assay (cTSA) demonstrated distinct thermal stabilization of IGF2BP3 upon treatment with I3IN-002, confirming successful target engagement within the cellular environment. I3IN-002 displayed an IC_50_ of approximately 2 μM in SEM-WT cells and modulated IGF2BP3-dependent cellular phenotypes. Further studies showed that I3IN-002 phenocopies several effects of genetic IGF2BP3 depletion, including alterations in metabolic and epitranscriptomic programs [67]. In leukemia cells, treatment reduced glycolytic activity, as indicated by decreased extracellular acidification rate and lactate production, and affected key metabolic intermediates, including lactate and fructose-1,6-bisphosphate. In addition, reductions in metabolites involved in one-carbon metabolism, such as S-adenosyl methionine, were reported. Consistently, global m^6^A RNA methylation levels were decreased, supporting a functional link between IGF2BP3 activity and epitranscriptomic regulation. Mechanistically, I3IN-002 was shown to reduce IGF2BP3 binding to target mRNAs and downregulate downstream protein expression, supporting interference with IGF2BP3-mediated post-transcriptional regulation.

Despite these encouraging findings, important limitations remain. Direct biophysical evidence of compound–protein binding is still lacking, and structure–activity relationships remain poorly defined, with only a small fraction of TR-FRET-active derivatives showing cellular activity. In addition, selectivity against other IGF2BP family members and RNA-binding proteins has not been systematically evaluated. Overall, while I3IN-002 represents an important proof-of-concept for pharmacological modulation of IGF2BP3-dependent programs, further studies are required to confirm target engagement and optimize potency and selectivity.

#### 5.1.2. AE-848 as a Potential Modulator of IGF2BP3

AE-848 was identified through a structure-based virtual screening approach targeting the RRM12 domain of IGF2BP3 (PDB ID: 6GX6) and subsequently evaluated in preliminary biological assays [58]. Available evidence suggests that AE-848 exerts anti-tumor effects in ovarian cancer models, where it inhibits cell proliferation, migration, and invasion in vitro. Mechanistically, AE-848 treatment was associated with reduced expression of key IGF2BP3 downstream targets, including *c-MYC*, *CDK2*, *CDK6*, *VEGF*, and *STAT1* [58], suggesting a potential modulation of IGF2BP3-dependent RNA regulatory networks. In vivo, AE-848 delayed tumor growth in subcutaneous ovarian cancer xenograft models. Consistently, immunohistochemical analyses showed decreased expression of c-MYC, CDK2, and VEGF in treated tumors, further supporting a potential impact on IGF2BP3-associated signaling pathways. In addition, AE-848 was reported to modulate the tumor immune microenvironment. Specifically, flow cytometry analyses indicated an increase in M1-like macrophage populations in vivo, while co-culture experiments suggested that AE-848 may impair M2-like polarization of tumor-associated macrophages. This effect was proposed to occur, at least in part, through downregulation of c-MYC signaling in tumor cells, thereby contributing to enhanced anti-tumor immune activity [58].

Despite these promising observations, several limitations remain. The chemical synthesis and experimental preparation of AE-848 have not been reported, limiting reproducibility and independent validation. Moreover, its proposed interaction with IGF2BP3 is based solely on docking studies targeting the RRM12 domain, without orthogonal biophysical or biochemical confirmation of direct binding. In addition, selectivity against other RBPs or related family members has not been investigated. Overall, AE-848 represents a preliminary bioactive scaffold with potential to modulate IGF2BP3-associated oncogenic pathways. However, further studies are required to confirm direct target engagement, define its mechanism of action more precisely, and establish its selectivity and drug-like properties.

#### 5.1.3. Small-Molecule Modulators of IGF2BP Proteins

The small molecule 7773 represents the first medicinal chemistry effort to directly target IGF2BP RNA-binding activity, emerging from a fluorescence polarization high-throughput screen (~27,000 compounds) designed to disrupt IGF2BP1–*KRAS* RNA interactions [66]. Biochemical validation using microscale thermophoresis (MST) confirmed direct binding to IGF2BP1 with a Kd of ~17 μM, consistent with an IC_50_ of ~30 μM for inhibition of RNA binding. Importantly, NMR (^15^N-HSQC) titration experiments provided residue-level insight, revealing that 7773 binds preferentially to the KH3–KH4 di-domain, inducing significant chemical shift perturbations in residues located at the inter-domain interface [66]. Notably, 7773 also binds IGF2BP3, despite having weaker affinity (Kd ~52 μM), and biochemical assays (MST and electrophoretic mobility shift assay, EMSA) confirm partial inhibition of IGF2BP3–RNA binding, highlighting limited selectivity (selectivity index, SI _IGF2BP1/IGF2BP3_ ~ 3) within the family. Subsequent medicinal chemistry optimization led to the identification of AVJ16, a derivative with improved potency [115]. Structure–activity relationship (SAR) studies retained the binding mode at the KH3–KH4 interface, but enhanced hydrophobic complementarity, resulting in an approximately 12-fold improved affinity [115]. Biophysical characterization using NMR and MST again confirmed direct interaction with IGF2BP1, while cellular target engagement was demonstrated through CETSA assays, where AVJ16 increased the thermal stability of IGF2BP1 (ΔTm ≈ +2 °C) [116]. No significant thermal shift was observed for IGF2BP2 or IGF2BP3, suggesting improved selectivity relative to 7773 despite the high homology across paralogs. In addition, AVJ16 prevented tumor growth in vivo, and induced cell death in human organoids, demonstrating its potential against IGF2BP1-expressing tumors. Together, these studies illustrate how integrated structural and biophysical approaches can elucidate the molecular determinants of IGF2BP inhibitor recognition, supporting progression toward a more refined lead candidate.

The fragment-based campaign reported in [117] combines biochemical screening, NMR, and structure-guided docking to characterize early binding modes of small molecules targeting IGF2BP2. Following fluorescence polarization screening, compound 4 emerged as the most potent hit, exhibiting inhibition of IGF2BP2–RNA interactions in the low-to-mid micromolar range. Ligand binding was validated by STD-NMR experiments that provided key insights into its binding mode. The benzoic acid moiety of 4 acts as the primary anchoring group, indicating close proximity to the protein surface. The central aromatic ring exhibits intermediate interaction, while the terminal phenyl group appears largely solvent-exposed, consistent with weaker STD signals. Then, docking studies were performed on both the KH3–KH4 domain and a homology model of the RRM1 domain, built using the homologous IGF2BP3 RRM12 structure, and assuming an RNA-competitive mechanism. In both models, the ligand carboxylate forms salt bridges with basic residues—Arg576 and Lys583 in KH34, and Arg90 in RRM1—mimicking interactions typically established by RNA phosphate groups [117]. The remaining scaffold occupies a shallow, partially hydrophobic groove overlapping the RNA-binding interface. Both docking poses remain speculative, and the available data do not permit a definitive assessment of their accuracy beyond their consistency with the STD-NMR observations. Nevertheless, the collected data support a competitive binding mode at the IGF2BP KH domains. High-resolution structural methods, such as X-ray crystallography, will be essential to validate these models and enable structure-guided optimization in future studies.

Table 2 summarizes the data on direct IGF2BP3-targeting compounds discussed in Section 5.1.

### 5.2. Rewiring IGF2BP3 Expression and Function Through Indirect Therapeutic Strategies

**Figure 5:**
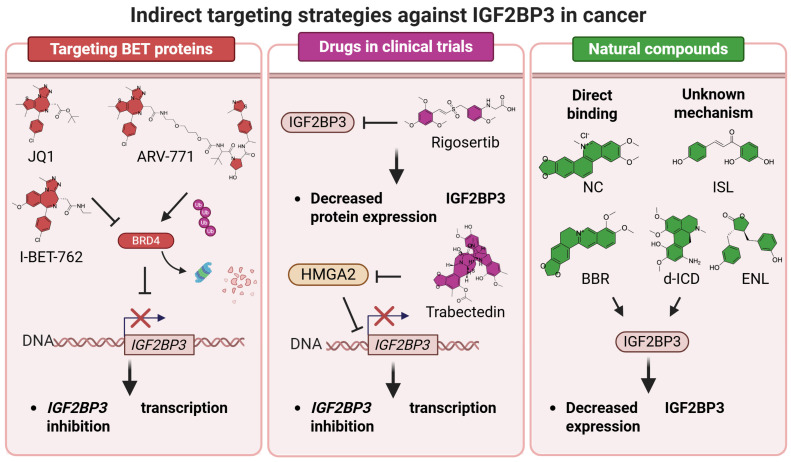
Schematic representation of indirect targeting of IGF2BP3 in cancer. Each panel shows the compound structure and the proposed mechanism leading to IGF2BP3 modulation. BET protein inhibitors and degraders (e.g., JQ1, I-BET-762, ARV-771) suppress *IGF2BP3* expression by disrupting BRD4-dependent super-enhancer activity at the *IGF2BP3* locus. Clinically relevant agents, including rigosertib and trabectedin, indirectly modulate IGF2BP3 activity through post-translational and transcriptional mechanisms, respectively, leading to destabilization of IGF2BP3-regulated mRNA networks. Natural compounds such as nitidine chloride (NC), berberine (BBR), isoliquiritigenin (ISL), isocorydine derivatives (d-ICD), and enterolactone (ENL) affect IGF2BP3 expression or function through diverse and not fully characterized mechanisms, ultimately resulting in reduced IGF2BP3 activity in cancer models. Created in BioRender. Mancarella, C. (2026) https://BioRender.com/ozqqis0.

Compared with direct inhibition, indirect targeting strategies offer a broader and potentially more tractable approach to modulating IGF2BP3 oncogenic activity. Rather than interfering with the highly dynamic RNA-binding interface, these approaches aim to rewire IGF2BP3 expression, stability, or downstream signaling through the modulation of upstream regulators, epigenetic programs, m^6^A machinery, and associated oncogenic pathways. Although several compounds and natural products have shown promising anticancer activity through indirect suppression of IGF2BP3-related networks, their mechanisms of action are often pleiotropic and incompletely defined. Consequently, disentangling IGF2BP3-specific effects from broader cellular responses remains a major challenge for the development of selective and mechanistically validated therapeutic strategies. A schematic representation of the compounds, which will be discussed below, is shown in Figure 5.

#### 5.2.1. Targeting BET Proteins to Epigenetically Regulate *IGF2BP3*

Bromodomain and extra-terminal domain (BET) proteins—including BRD2, BRD3, BRD4, and BRDT—constitute a family of epigenetic regulators that operate at the interface between chromatin modifications and transcriptional activation. These proteins contain two tandem bromodomains that recognize acetylated lysine residues on histones, thereby promoting transcription through recruitment of the Mediator complex and the positive transcription elongation factor b. Although BET proteins are essential for normal cellular homeostasis, their dysregulation has been implicated in cancer, where BRD2, BRD3, and BRD4 activate gene programs involved in cell cycle progression, apoptosis resistance, inflammation, DNA repair, and senescence-associated transformation [118].

Accordingly, multiple inhibitors have been developed to block BET activity by competitively displacing bromodomain proteins from acetylated histones. Several of these compounds, such as I-BET-762 [119], are being evaluated in clinical trials and are structurally related to the prototypical BET inhibitor—the thienotriazolodiazepine JQ1. Novel chemical strategies aimed at promoting proteasome-mediated degradation of BET proteins and eliciting a potent antiproliferative response provided the so-called PROteolysis TArgeting Chimeras (PROTACs) which are now in advanced preclinical/early clinical stages, as discussed in recent reviews [120,121].

Accumulating evidence indicates that BET inhibition modulates IGF2BP3 expression in cancer. *IGF2BP3* has been identified as a direct transcriptional target of BRD4 across multiple tumor models [122]. Its expression is driven by super-enhancer activity, as demonstrated by ChIP assays in acute myeloid leukemia cells showing strong enrichment of H3K27ac, H3K4me1, and BRD4 at regulatory regions upstream of the *IGF2BP3* transcription start site [122,123]. Consistently, siRNA-mediated knockdown studies demonstrated that BRD4 is the primary BET family member regulating *IGF2BP3* expression [123].

Pharmacological inhibition of BET proteins reduces IGF2BP3 levels across multiple cancer models. The BET inhibitor JQ1 downregulates IGF2BP3 expression in neonatal megakaryocytes, leukemic cells, and Ewing sarcoma models [47,68,124]. Similarly, both JQ1 and the PROTAC ARV-771 significantly decrease IGF2BP3 expression in prostate cancer cell lines (LNCaP, PC3, and DU145), accompanied by loss of BRD4 occupancy at the *IGF2BP3* promoter [125]. Importantly, BET inhibition also reduces the expression of IGF2BP3 downstream targets, including *ABCF1*, *MMP9*, *ABCG2*, and *DDX21*. PROTAC-based strategies appear to exert even stronger effects. The BET degrader dBET1 induces a dose-dependent reduction in IGF2BP3 protein levels in Merkel cell carcinoma models, showing greater efficacy than JQ1 at comparable concentrations [123].

Collectively, these findings highlight that both BET inhibition and degradation represent indirect yet effective strategies to suppress IGF2BP3, targeting a regulatory axis at the intersection of super-enhancer activity and m^6^A-dependent post-transcriptional control. Functionally, BET modulation reduces tumor cell proliferation in vitro and exerts antitumor activity in vivo.

#### 5.2.2. Cancer Therapies in Clinical Trials Indirectly Targeting IGF2BP3

Rigosertib (ON-01910.Na) and trabectedin are clinically relevant anticancer agents with distinct mechanisms that converge on the suppression of oncogenic programs, including IGF2BP3 downregulation.

Rigosertib is a benzyl styryl sulfone multi-kinase inhibitor initially developed as a Plk1 inhibitor and later characterized as a Ras-mimetic that disrupts Ras–Raf interactions and inhibits PI3K/Akt signaling. In a high-throughput screen of FDA-approved compounds targeting RNA-binding proteins, rigosertib emerged as a potent and selective inhibitor of IGF2BP3 in lung adenocarcinoma cells [126]. Multi-omics analyses, including eCLIP-seq, RNA-seq, and m^6^A-seq, identified IGF2BP3 as a critical regulator of transcripts involved in ferroptosis suppression and tumor metabolism. Treatment with rigosertib significantly decreased IGF2BP3 protein levels, destabilizing oncogenic mRNAs normally stabilized by IGF2BP3, such as *MYC*, *MET*, and *ABC* transporter family members. Functionally, this led to increased sensitivity of cancer cells to ferroptosis inducers, enhanced lipid peroxidation, and selective impairment of tumor proliferation, sparing normal cells. In xenograft models, systemic rigosertib treatment reduced tumor growth while decreasing intratumoral IGF2BP3 expression, providing preclinical evidence that targeting IGF2BP3 contributes to its antitumor activity [126]. These findings establish rigosertib as an indirect post-transcriptional IGF2BP3 modulator, linking upstream kinase inhibition with disruption of m^6^A-dependent oncogenic RNA networks.

Trabectedin is a marine-derived tetrahydroisoquinoline alkaloid that binds the minor groove of DNA, inducing DNA bending, transcriptional interference, and activation of DNA damage response pathways. Trabectedin also remodels the tumor microenvironment by selectively depleting tumor-associated macrophages and reducing pro-inflammatory cytokines, contributing to antitumor efficacy in soft tissue sarcomas and relapsed ovarian cancer [127]. Trabectedin was shown to displace HMGA proteins from HMGA-responsive promoters, impairing their transcriptional activity [128]. Among these targets, HMGA2, a chromatin architectural factor and oncogenic driver, has been shown to sustain *IGF2BP3* transcription in cancer cells. Treatment with trabectedin did not modify HMGA2 protein expression levels but did affect the expression of the HMGA2 targets I*GF2BP2* and *IGF2BP3*, resulting in modulated expression and downregulation of the IGF2BP3 downstream IGF2/IGF1R signaling pathway, thereby impairing proliferation, migration, and survival of tumor cells [127]. This highlights a dual layer of antitumor action: direct transcriptional perturbation and indirect post-transcriptional suppression of IGF2BP3-dependent oncogenic networks.

Overall, available preclinical and clinical evidence supports the concept that IGF2BP3 can be indirectly modulated by clinically relevant anticancer agents, including rigosertib, which targets kinase and Ras-dependent signaling, and trabectedin, which exerts transcriptional and chromatin-level effects. However, the anticancer activity of these compounds is likely multifactorial, with IGF2BP3 representing one of several converging downstream nodes contributing to their biological effects.

#### 5.2.3. Natural Compounds with Unexpected Effects on IGF2BP3 Expression and Function

Several natural compounds have recently emerged as potential modulators of IGF2BP3 expression and activity in cancer cells. Despite their structural diversity, these compounds converge on common mechanisms involving m^6^A-dependent RNA regulation, protein destabilization and degradation, and suppression of oncogenic signaling pathways. Elucidating the molecular basis of these effects may facilitate the rational development of the next generation of indirect IGF2BP3 modulators.

Nitidine chloride (NC) is a benzo[c]phenanthridine alkaloid extracted from the roots and stems of *Zanthoxylum nitidum* (Roxb.). Early studies mainly focused on its antibacterial, anti-inflammatory, and antimalarial activities; however, increasing evidence has highlighted its anti-tumor potential. NC exhibits inhibitory effects against multiple tumor types [129] through several mechanisms, including disruption of mitotic nuclear division, chromosome segregation, and microtubule-associated processes. In colorectal cancer, NC was shown to bind KIF20A with high affinity, thereby suppressing tumor cell proliferation [130,131]. Interestingly, molecular docking and molecular dynamics simulations also indicated a stable interaction between NC and IGF2BP3, suggesting the possibility that IGF2BP3 may directly interact with NC [132]. In hepatocellular carcinoma models, NC treatment reduced IGF2BP3 expression, resulting in decreased cell proliferation in vitro and suppression of tumor growth and metastasis formation in zebrafish models. Integrated MeRIP-seq and RIP-seq analyses further identified 197 IGF2BP3-associated transcripts that were simultaneously downregulated at both mRNA and m^6^A levels following NC treatment, with significant enrichment in metabolism-related pathways. Collectively, these findings suggest that NC may exert part of its anti-tumor activity through m^6^A-dependent regulation of IGF2BP3-associated metabolic programs.

Berberine (BBR), one of the most important active ingredients in *Coptidis Rhizoma*, has various pharmacological activities such as anti-tumor and anti-inflammatory activities [133]. Several studies have shown that BBR downregulates IGF2BP3 in tumor cells [133,134,135]. Mechanistically, BBR down-regulated IGF2BP3 expression and inhibited colorectal cancer growth in mice. CETSA and DARTS analyses suggested a direct interaction between BBR and IGF2BP3. BBR may induce conformational changes in IGF2BP3, thereby reducing its cytoplasmic stability. Mechanistically, BBR promoted TRIM21-mediated ubiquitination of IGF2BP3. RIP assays further demonstrated that BBR inhibited IGF2BP3-mediated stabilization of *CDK4*/*CCND1* mRNAs, thereby promoting G1/S phase arrest in colorectal cancer cells.

Isoliquiritigenin (ISL), derived from the Chinese herb licorice, exhibits anti-tumor activity in multiple human cancers [136]. Recent findings indicate that ISL exerts anti-tumor effects in NSCLC through modulation of the IGF2BP3/m^6^A/TWIST1 axis [136]. Mechanistically, ISL reduced m^6^A modification and down-regulated IGF2BP3 expression in NSCLC. Furthermore, IGF2BP3 enhanced the mRNA stability of twist family bHLH transcription factor 1 (TWIST1) in an m^6^A-dependent manner. ISL treatment combined with TWIST1 knockdown effectively reversed IGF2BP3 overexpression-induced NSCLC cell proliferation, migration and invasion [136].

The use of an isocorydine derivative (d-ICD), i.e., an alkaloid derivative isolated from plants of the *Papaveraceae* family, has been demonstrated to inhibit IGF2BP3 expression and reduce the growth of hepatocellular carcinoma cells [48].

Enterolactone (ENL) has demonstrated anti-tumor activity in several human cancers. In epithelial ovarian cancer, ENL reduced IGF2BP3 expression, thereby suppressing the VEGF/PI3K/AKT signaling pathway and inhibiting tumor cell proliferation, migration, invasion, and angiogenesis. Because ENL is a mammalian lignan produced via the biotransformation of dietary plant lignans by intestinal bacteria, its therapeutic action is intimately linked to the host’s digestive ecology. Consequently, ENL partially restored gut microbiota composition both in terms of reversing tumor-induced dysbiosis by reducing harmful *Bacteroidetes* and promoting beneficial strains, e.g., *Alistipes*, particularly in combination with short hairpin RNA targeting *IGF2BP3* [137].

Natural compound mechanisms of action are often not fully defined and may extend beyond modulation of IGF2BP3-related pathways. This is particularly relevant forBBR and its derivatives, which are known to interact with multiple cellular targets. Consequently, their observed anticancer effects cannot be exclusively attributed to IGF2BP3 regulation, and additional molecular targets and signaling pathways are likely involved.

Table 3 summarizes the data on indirect IGF2BP3 modulators discussed in Section 5.2.

## 6. Critical Issues

Although IGF2BP3 has been extensively implicated in tumor initiation and progression, and several compounds have emerged for its direct or indirect targeting, more comprehensive preclinical studies are required before IGF2BP3-targeted therapies can be considered a realistic clinical possibility:

- Clinical implementation of IGF2BP3 as a diagnostic or prognostic biomarker remains challenging. Optimal detection methods, scoring systems, and clinically standardized cut-offs still need to be established before its translation into routine clinical practice [138,139]. Moreover, intratumoral heterogeneity and antibody cross-reactivity with other IGF2BP family members may affect the interpretation of IGF2BP3 expression data, particularly in tumors co-expressing multiple paralogs [41]. Prospective studies using harmonized analytical approaches will be required to define the clinical utility of IGF2BP3 as a biomarker.
- Predictive biomarkers of response to IGF2BP3-targeted therapies need to be identified. Although IGF2BP3 expression itself represents the best candidate, future studies should determine whether integrating IGF2BP3 expression with molecular features, including m^6^A epitranscriptomic profiles, expression of IGF2BP3 target transcripts, and co-expression of key protein partners, can identify tumors that are more likely to benefit from IGF2BP3-targeted therapies.
- The similarity among IGF2BP paralogs, together with the dynamic and multivalent nature of IGF2BP–RNA interactions, impairs the development of compounds capable of achieving complete paralog selectivity. The absence of high-resolution structures of IGF2BP3–RNA complexes further limits structure-guided drug design approaches. As a consequence, potential cross-reactivity of IGF2BP3 inhibitors with related IGF2BP family members remains an important issue that requires careful evaluation. Since IGF2BP1 and IGF2BP2 contribute to distinct physiological and cancer-associated RNA regulatory networks, concomitant inhibition of these paralogs may influence both therapeutic efficacy and safety profiles. A deeper understanding of paralog-specific biology, target selectivity, and context-dependent functions will therefore be essential for the development of selective IGF2BP-targeted therapies.
- Resistance to either direct or indirect IGF2BP3-targeting strategies has not yet been specifically investigated. While this likely reflects the early stage of development of direct anti-IGF2BP3 agents, studies on indirect approaches have also not addressed resistance in the specific context of IGF2BP3 blockade, although resistance mechanisms to the individual targeted pathways have been extensively characterized [140,141,142,143,144]. Moreover, it remains unknown whether tumor cells exploit the functional redundancy among IGF2BP family members to sustain oncogenic signaling upon IGF2BP3 depletion. Elucidating these adaptive mechanisms will be crucial for anticipating resistance mechanisms and guiding the development of effective targeted therapies.
- Comprehensive studies are required to define the safety profile of IGF2BP3-targeted therapies. The restricted expression of IGF2BP3 in most adult tissues, together with its re-expression in many cancers, suggests a potentially favorable therapeutic window. Encouragingly, genetic deletion of Igf2bp3 does not impair normal hematopoiesis in transgenic mice [72,145]. However, potential adverse effects on reproductive functions should be carefully evaluated, as Igf2bp3 knockout mice exhibit defective spermatogenesis and male subfertility or infertility [146], and IGF2BP3 has been implicated in placental development through regulation of trophoblast proliferation, migration, and invasion [147]. Although these findings derive primarily from germline genetic models rather than therapeutic inhibition in adults, they identify fertility and pregnancy as important areas for future preclinical safety assessment. In addition, the potential consequences of inhibitor cross-reactivity with IGF2BP1 and IGF2BP2 with respect to adverse effects remain to be elucidated.

## 7. Conclusions and Future Perspectives

Preclinical and clinical evidence support the role of IGF2BP3 as a central post-transcriptional regulator linking epitranscriptomic to key oncogenic processes, including proliferation, migration, metabolic responses, and immune evasion. The pleiotropic effects associated with IGF2BP3 reflect its integration within extensive regulatory networks involving multiple upstream modulators and downstream interactors. Its aberrant expression in cancer arises through diverse mechanisms, including DNA hypomethylation, dysregulated transcription factors and non-coding RNAs, altered ubiquitin–proteasome system activity, and, less frequently, gene amplification. In turn, IGF2BP3 regulates RNA fate by recognizing m^6^A-modified transcripts and engaging dynamic interactions with miRNA machinery, stress-response proteins, ubiquitination systems, and cytoskeletal transport complexes, thereby controlling RNA stability, localization, and translation.

Despite the extensive body of biological research, IGF2BP3 remains a challenging target from a drug discovery perspective. The current landscape is characterized by a strong imbalance between extensive biological characterization and relatively limited medicinal chemistry efforts. Most reported small molecules originate from phenotypic or virtual screening campaigns, often followed by partial optimization supported by biophysical validation assays, rather than from structure-driven design. This reflects both the early stage of the field and the intrinsic difficulty of targeting multivalent RNA–protein interactions.

At present, direct and indirect strategies should be viewed as complementary rather than competing approaches. Direct inhibition offers the prospect of selectively disrupting IGF2BP3–RNA interactions and, therefore, greater mechanistic specificity. However, current direct inhibitors should be considered hit or early lead compounds that require substantial medicinal chemistry optimization to improve potency, selectivity, and drug-like properties. A central challenge is the high conservation of the RRM and KH domain architecture and the approximately 63–74% sequence identity among IGF2BP1, IGF2BP2, and IGF2BP3, which create a substantial risk of cross-paralogue activity. Nevertheless, differences in developmental and tumor-specific expression, RNA-sequence and motif-spacing preferences, non-conserved loops and linker regions, domain dynamics, and cancer dependencies may provide exploitable determinants for selective ligand design. Although chemical modulators have now been reported for all three family members, systematic cross-paralogue profiling remains limited, preventing definitive conclusions regarding their relative druggability. Future discovery programs should, therefore, evaluate candidate IGF2BP3 ligands against IGF2BP1 and IGF2BP2 using matched biochemical, biophysical, and cellular assays, while also determining whether selective IGF2BP3 inhibition or broader pan-IGF2BP activity provides the most favorable efficacy–toxicity balance in each tumor context. In contrast, indirect strategies, including BET inhibition and degradation, epitranscriptomic modulation, and natural products, are pharmacologically more mature, with compounds already in clinical development. Their major limitation is their multiple mechanisms of action, which complicate the attribution of therapeutic effects specifically to IGF2BP3 modulation. Nevertheless, early proof-of-concept compounds targeting IGF2BP–RNA interactions, together with indirect strategies demonstrate that pharmacological intervention is feasible. Importantly, the integration of AlphaFold structural prediction, biophysical approaches, and multi-omics technologies is progressively improving our understanding of IGF2BP3 recognition mechanisms, providing a foundation for rational drug design. Among emerging therapeutic modalities [148], targeted protein degradation, particularly via PROTAC technology, represents a promising approach for addressing difficult-to-drug RBPs. PROTACs enable ubiquitin-dependent elimination of target proteins through induced proximity with E3 ligases. Successful degradation of RBPs, such as HuR [149], LIN28, and RBFOX1 [150], highlights the tractability of this approach. In parallel, RNA-based degraders (e.g., ORN3P1 targeting Lin28A) [150], further expand the chemical space of proximity-driven modalities [151]. IGF2BP3 is also linked to ubiquitin regulation via E3 ligases, including Parkin and MKRN2 [55,100], supporting its potential amenability to future degradation strategies.

Epitranscriptomic modulation of m^6^A signaling offers an additional indirect yet chemically actionable route to regulate IGF2BP3 function. Although no m^6^A-targeting drugs are clinically approved, inhibitors of METTL3 and FTO are under investigation [152,153]. Because IGF2BP3 stabilizes m^6^A-modified transcripts, perturbation of methylation dynamics can indirectly attenuate its oncogenic output. The METTL3 inhibitor STM2457, for instance, disrupts IGF2BP3-dependent stabilization of metabolic transcripts, thereby impairing tumor adaptation and enhancing sensitivity to therapy [154]. Consistently, combined targeting of METTL3 and IGF2BP3 further suppresses oncogenic signaling [155], while additional compounds such as celastrol reinforce the therapeutic relevance of this axis [156,157].

RNA-based therapeutic approaches provide further opportunities to modulate IGF2BP3 expression and function. These include siRNAs, antisense oligonucleotides (ASOs), and emerging RNA therapeutics already validated clinically in other disease contexts [158]. A DDX5 mRNA-targeting ASO has recently emerged as a promising strategy in castration-resistant prostate cancer, given that DDX5 regulates mRNA stability via interacting with the IGF2BP complex, thereby modulating the expression of downstream target oncogenes [159]. IGF2BP3 is also regulated by tumor-suppressive miRNAs (e.g., miR-34a), while oncogenic lncRNAs and circRNAs networks sustain its expression. Restoring miRNA activity or disrupting these regulatory circuits represents a complementary indirect strategy for IGF2BP3 suppression. In addition, IGF2BP3 has been explored as a tumor-associated antigen in vaccine-based approaches, with evidence of immunogenicity in patient-derived samples and early translational studies [160,161,162], supporting its broader potential in immunotherapy.

Overall, IGF2BP3 exemplifies how advances in RNA biology, epitranscriptomics, and emerging chemical modalities are converging to transform challenging RBPs into increasingly tractable targets. Future progress will depend on expanding the current small-molecule toolkit, integrating innovative chemical modalities, and systematically translating screening-derived hits into well-characterized lead compounds. Ultimately, these efforts will be essential to determine whether IGF2BP3 can be fully validated as a truly actionable therapeutic target in oncology.

## Figures and Tables

**Table 1:** Selection of IGF2BP3 mRNA targets.

Target mRNA	Binding Site	Regulation of Target mRNA	Reference
*ABCF1*	n.a.	*ABCF1* mRNA serves as a decoy for IGF2BP3, preventing the protein from binding to its oncogenic RNA targets	[47]
*ABCG2*	n.a.	mRNA stabilization	[48,49]
*ARF6*	n.a.	mRNA localization	[50]
*ARHGEF4*	n.a.	mRNA localization	[50]
*CCND1*	3′-UTR; CDS	mRNA stability; protection from microRNA-dependent repression	[51,52,53]
*CCND3*	3′-UTR	mRNA stability; protection from microRNA-dependent repression	[53]
*CCNG1*	3′-UTR	mRNA stability; protection from microRNA-dependent repression	[53]
*CD44*	3′-UTR	mRNA stability	[54,55]
*CD164*	n.a.	Translation enhancement	[56,57]
*CDK2*	n.a.	Translation enhancement	[58]
*CDK6*	5′-UTR	mRNA stability via m^6^A	[59]
*EIF4EBP2*	3′-UTR	mRNA degradation	[60]
*GPX4*	CDS; 3′-UTR	mRNA stability and translation	[61]
*HMGA2*	3′-UTR	Prevention of miRNA-directed mRNA decay	[62]
*IGF1R*	3′-UTR	mRNA stability	[63,64]
*IGF2*	5′-UTR	Translation enhancement	[65]
*KRAS*	n.a.	Translation enhancement	[66]
*LIN28B*	3′-UTR	Prevention of miRNA-directed mRNA decay	[62]
*MAT2B*	3′-UTR	Translation enhancement	[67]
*MYC*	3′-UTR	Translation enhancement	[68]
*PDPN*	3′-UTR	mRNA stability	[55]
*SNAI2*	5′-UTR	Translation enhancement	[69]
*STAT1*	n.a.	mRNA stability	[58]
*TP53*	3′-UTR	mRNA degradation	[70]
*VEGF*	n.a.	mRNA stability	[71]

n.a., not available.

**Table 2:** Direct IGF2BP-targeting compounds.

Compound	Target Region	Mechanism	Biophysical/ Validation Techniques	Kd/IC_50_ (IGF2BP1/2/3)	In Vivo Tumor Model Outputs	Reproducibility Constraints	Reference
I3IN-002	IGF2BP3 RRM12 (PDB: 6GX6)	Competitive RNA disruption	Virtual screening, TR-FRET, TSA, CTSA cell assays	Increase IGF2BP3 melting temperature (*T_m_*)	↓ leukemia metabolic activity and m^6^A levels in a syngeneic transplantation C57BL/6J mouse model	no selectivity data	[113,114]
AE-848	IGF2BP3 RRM12 (docking-based)	Non-validated IGF2BP3 binding (Possible indirect modulation)	In silico docking, cell assays, in vivo xenografts	NR	↓ ovarian xenograft growth and c-MYC/CDK2/VEGF expression; increased M1 macrophages	Limited SAR; docking-based data	[58]
7773	IGF2BP1 KH3–KH4 (±IGF2BP3)	Weak RNA competition	FP screening, MST, EMSA, ^15^N-HSQC NMR	IGF2BP1 Kd ≈ 17 μM; RNA inhibition IC_50_ ≈ 30 μM; IGF2BP3 Kd ≈ 52 μM; IGF2BP2: NR	NR	Low potency; partial IGF2BP3 inhibition; limited selectivity	[66]
AVJ16	IGF2BP1 KH3–KH4	Competitive RNA disruption	FP, MST, ^15^N-HSQC NMR, CETSA, cell assays, in vivo	IGF2BP1 K_d_ = 1.4 μM; IGF2BP2/3: same as 7773	↓ tumor growth and organoid viability in IGF2BP1-driven models	Mainly IGF2BP1-focused; limited paralog profiling	[115,116]
Compound 4	IGF2BP2 KH3–KH4 (±RRM1 model)	Weak RNA competition	FP screening, STD-NMR, docking (KH3–KH4/RRM1 homology model)	IC_50_ ≈ 81 μM IGF2BP2–RNA inhibition; IGF2BP1/3: NR	↓ tumor growth in a zebrafish embryo xenograft model	Computational binding model; low potency	[117]

NR, not reported.

**Table 3:** Indirect IGF2BP3-targeting compounds: epigenetic, clinical, and natural modulators of IGF2BP3 expression and activity.

Compound	Target Region	Mechanism	Biophysical/ Validation Techniques	In Vivo Tumor Model Outputs	Reproducibility Constraints	Reference
JQ1	BET (BRD2/3/4 bromodomains)	Chromatin displacement (BET inhibition)	ChIP (H3K27ac/ BRD4), transcriptomics	↓ IGF2BP3 expression; reduced xenograft growth	Indirect IGF2BP3 regulation; context-dependent response	[47,68,124]
I-BET-762	BET bromodomains	Chromatin displacement	Bromodomain binding assays, gene expression profiling	Antitumor activity reported; IGF2BP3-specific effects NR	IGF2BP3 effects require cellular validation	[119]
ARV-771	BRD4	Protein degradation (PROTAC)	PROTAC degradation assays, ChIP	↓ IGF2BP3 expression; inhibited xenograft growth	Depends on E3 ligase expression and cellular context	[125]
dBET1	BET proteins	Targeted degradation	Western blot, degradation kinetics	Dose-dependent IGF2BP3 degradation; stronger suppression than JQ1	Pharmacokinetic and degradation limitations	[123]
Rigosertib	IGF2BP3 RNA network (indirect)	Post-transcriptional network disruption (Ras/PI3K-linked)	eCLIP-seq, RNA-seq, m^6^A-seq, xenografts	↓ Tumor growth; ↑ ferroptosis sensitivity	Multi-target mechanism complicates IGF2BP3 attribution	[126]
Trabectedin	DNA minor groove/HMGA axis	Transcriptional interference → IGF2BP3 suppression	ChIP, transcriptomics, DNA-binding assays	Antitumor activity in sarcoma/ovarian models	Complex DNA damage, immune, and transcriptional effects	[127]
Nitidine chloride (NC)	IGF2BP3 axis (putative)	Expression suppression/RNA program disruption	Docking, MD simulations, MeRIP-seq, RIP-seq	↓ HCC growth and metastasis (zebrafish)	Direct binding requires biochemical confirmation	[130,131]
Berberine (BBR)	IGF2BP3	Protein destabilization (ubiquitin-mediated)	CETSA, DARTS, RIP, ubiquitination assays	↓ CRC growth; reduced IGF2BP3 stability	Pleiotropic effects; IGF2BP3 contribution unclear	[133,134,135]
Isoliquiritigenin (ISL)	IGF2BP3/m^6^A/TWIST1 axis	Epitranscriptomic suppression	m^6^A profiling, rescue assays	↓ NSCLC proliferation, migration, and invasion	m^6^A-dependent mechanism; no direct binding evidence	[136]
d-ICD (isocorydine derivative)	IGF2BP3	Expression suppression	In vivo tumor models, proliferation assays	↓ HCC growth in vivo	Limited mechanistic validation	[48]
Enterolactone (ENL)	IGF2BP3 → VEGF/PI3K/AKT axis	Pathway suppression	Expression profiling, microbiome analysis	↓ Ovarian tumor growth, invasion, angiogenesis	Microbiome and indirect pathway effects complicate interpretation	[137]

## Data Availability

No new data were created or analyzed in this study. Data sharing is not applicable to this article.

## Abbreviations

The following abbreviations are used in this manuscript:

AGO2	Argonaute 2
BBR	Berberine
BET	Bromodomain and extra-terminal domain
CCND1	Cyclin D1
CDK	Cyclin-dependent kinase
circRNA	Circular RNA
CETSA	Cellular thermal shift assay
ChIP	Chromatin immunoprecipitation
CLIP	Crosslinking immunoprecipitation
CRC	Colorectal cancer
DNA	Deoxyribonucleic acid
DARTS	Drug affinity responsive target stability
EMT	Epithelial–mesenchymal transition
EMSA	Electrophoretic mobility shift assay
eCLIP	Enhanced crosslinking and immunoprecipitation
ENL	Enterolactone
FDA	Food and Drug Administration
FTO	Fat mass and obesity-associated protein
G3BP1	Ras GTPase-activating protein-binding protein 1
HMGA1/2	High mobility group A1/2
IGF	Insulin-like growth factor
IGF1R	Insulin-like growth factor 1 receptor
IGF2	Insulin-like growth factor 2
IGF2BP1/2/3	Insulin-like growth factor 2 mRNA-binding protein 1/2/3
ISL	Isoliquiritigenin
KH domain	K homology domain
KIF20A	Kinesin family member 20A
lncRNA	Long non-coding RNA
MAPK	Mitogen-activated protein kinase
MAT2B	Methionine adenosyltransferase 2B
METTL3/METTL14	Methyltransferase-like 3/14
miRNA/miR	MicroRNA
MMP9	Matrix metalloproteinase 9
m^6^A	N6-methyladenosine
m^7^G	N7-methylguanosine
MST	Microscale thermophoresis
MYC/MYCN	MYC proto-oncogene/MYCN proto-oncogene
NF-κB	Nuclear factor kappa-light-chain-enhancer of activated B cells
NC	Nitidine chloride
NSCLC	Non-small cell lung cancer
PAR-CLIP	Photoactivatable ribonucleoside-enhanced crosslinking immunoprecipitation
PI3K/AKT	Phosphoinositide 3-kinase/protein kinase B pathway
PROTAC	PROteolysis TArgeting Chimera
RBP	RNA-binding protein
RISC	RNA-induced silencing complex
RNA	Ribonucleic acid
RNP	ribonucleoprotein
RRM	RNA recognition motif
RIP	RNA immunoprecipitation
seq	sequencing
STAT1	Signal transducer and activator of transcription 1
TAMs	Tumor-associated macrophages
TET3	Ten-eleven translocation methylcytosine dioxygenase 3
TME	Tumor microenvironment
TR-FRET	Time-resolved fluorescence resonance energy transfer
TWIST1	Twist family bHLH transcription factor 1
USP	Ubiquitin-specific protease
VEGF	Vascular endothelial growth factor
